# Echovirus 7 associated with hand, foot, and mouth disease in mainland China has undergone a recombination event

**DOI:** 10.1007/s00705-015-2350-1

**Published:** 2015-02-15

**Authors:** Xin Yao, Lian-Lian Bian, Qun-Ying Mao, Feng-Cai Zhu, Qiang Ye, Zheng-Lun Liang

**Affiliations:** 1 National Institute for Food and Drug Control, No. 2 Tiantanxili, Beijing, 100050 China; 2 Jiangsu Provincial Center for Disease Control and Prevention, Nanjing, 210009 China

## Abstract

To investigate the evolution of echovirus 7 (Echo7) strains and the relationship between Echo7 strains and the prototype strain Wallace, phylogenetic analysis of Echo7 strains prevailing in mainland China was performed. The Echo7 strain, DH22G/JS/2012 was isolated from a 32-month-old boy who was clinically diagnosed with HFMD. The complete genome sequence of this isolate was determined after the virus was propagated in cell culture. Phylogenetic analysis showed that the subgroups B1 and C1 prevailed in mainland China from 1998 to 2012 and that the subgroup B2 began to circulate in mainland China in 2009. The result of Simplot analysis showed that the Echo7 strain DH22G/JS/2012 is a recombinant coxsackievirus B4 (CVB4) that circulated in mainland China in 2010.

Echovirus 7 (Echo7) is a member of the family *Picornaviridae*, genus *Enterovirus* (EVs). The prototype, Echo7 strain Wallace, was isolated in the United States in 1953 [1]. Clinical presentations of Echo7 include aseptic meningitis, meningoencephalitis, and gastrointestinal manifestations [2]. Over the past few years, there have been reports of fatal neonatal Eco 7 infections in many countries [3, 4]. However, hand, foot, and mouth disease (HFMD) has rarely been reported to be associated with Echo7 infection [5]. To our knowledge, there has been no reported case of Echo7-infection-associated HFMD in mainland China.

To date, only five complete genome sequences of Echo7 are available in the GenBank database, including three for prototype Wallace strains (GenBank accession nos. AF465516, AY302559, and AY036579), one for the UMMC strain (AY036578), and one for the LR11F7 strain (LFJ460595). No complete sequence of Echo7 has been reported in mainland China. In addition, the propensity of Echo7 strains to evolve and the relationship between the prevailing Echo7 strain in mainland China and Wallace have not been studied. We first isolated an Echo7 strain (GenBank no. KJ765699), named DH22G/JS/2012, from a HFMD patient in mainland China in 2012. The complete nucleotide sequence of DH22G/JS/2012 was compared to that of strain Wallace. A phylogenetic tree was constructed, and Simplot analysis was carried out between DH22G/JS/2012 and HEV-B. Our results show that, despite being related to Wallace, Echo7 strains prevailing in mainland China are recombinant HEV-B strains.

The samples were collected during a phase III clinical trial of an inactivated enterovirus 71 (EV71) vaccine [6]. The study was approved by the institutional review broad of Jiangsu Provincial Center of Disease Control and Prevention and done in accordance with the Declaration of Helsinki, Good Clinical Practice, and Chinese regulatory requirements. All guardians of participants provided written informed consent. The DH22G/JS/2012 strain was isolated from a 32-month-old boy who was clinically diagnosed with HFMD. The clinical symptom was rash on hands and limbs. The boy recovered spontaneously 3 days later. Throat and rectal swabs were collected within 24 h after onset. A real-time PCR kit for detection of EVs (Jiangsu Melo Bioscience) was used for sample testing. The test results showed that the sample was positive for EVs and negative for EV71 and coxsackievirus A16 (CVA16). During the follow-up, serial throat and rectal swabs were collected until the patient had recovered fully.

The serial samples were tested for enteroviruses by PCR with primers targeting the 5′ untranslated region (5′-UTR) and the VP1 region, following the protocol described in previous studies [7, 8]. BLAST analysis of the 5′-UTR and VP1 sequences from the serial samples showed that the patient was infected with Echo7. After the virus was propagated in cell culture, overlapping fragments were sequenced using degenerate primers and were assembled to construct the complete genome sequence.

To better understand the molecular epidemiology of Echo7 in mainland China, phylogenetic analysis, using MEGA 5 program [9], was conducted based on the VP1 sequence of the global Echo7 strains. All Echo7 strains were classified into three groups (A, B, and C), with at least 13.7 % VP1 nucleotide diversity between each subgroup. Distance analysis showed that the nucleotide identities between A and B, between A and C, and between B and C were 78.2 %-82.5 %, 80.5 %-82.0 %, and 84.0 %-86.3 %, respectively. The Wallace strains were clustered in group A. Group B was further divided into three subgroups, B1, B2, and B3. The B1 subgroup included strains from mainland China and was divided into two clusters, consisting of isolates from mainland China and India, respectively. The B3 subgroup was formed by strains form India and Japan, with no Chinese Echo7 strains. All Chinese Echo7 strains fell into subgroups B1 and B2, and group C, representing isolates from 1998-2012, 2009-2010, and 1998-2010, respectively. Our results indicated that B1 and C subgroups prevailed in Mainland China during 1998-2012, and the B2 subgroup began to circulate in mainland China in 2009 (Fig. 1).

**Fig. 1:**
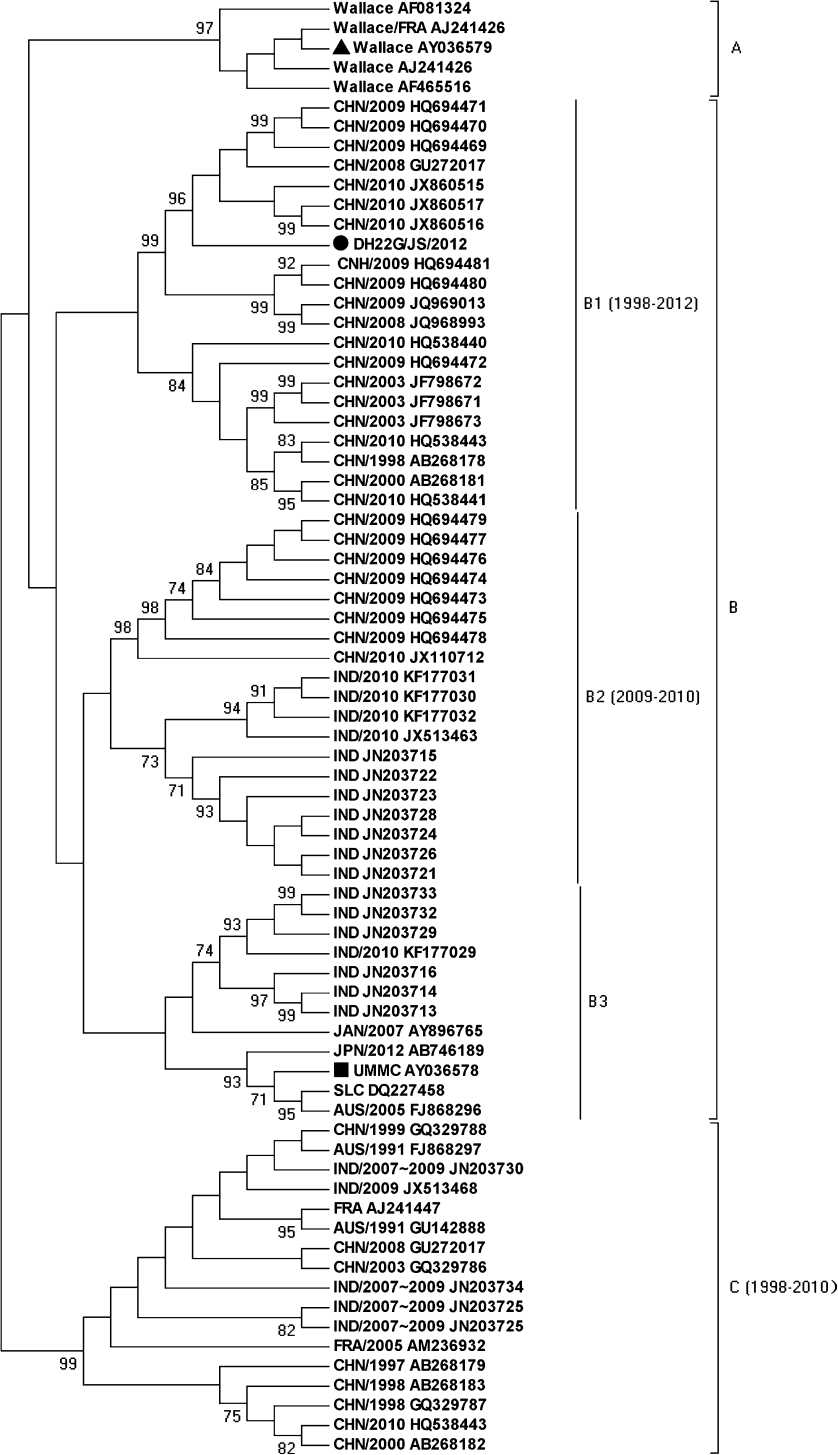
Phylogenetic tree based on partial VP1 sequences (647 bp) of Echo7 isolates of different geographic and temporal origin. The neighbor-joining method was used to construct the tree. The phylogenetic tree was determined for 1,000 replicates with random seeds. A circle indicates a B1 genotype strain isolated in China in 1999, a square indicates the DH22G/JS/2012 strain. Only strong bootstrap values (>70 %) are shown. AUS, Australia; CHN, China; FRA, France; IND, India; JPN, Japan; USA, United States

The complete genome sequence of DH22G/JS/2012 and five complete genome sequences of Echo7 were aligned using DNASTAR (version 7.10). We found that the five complete genome sequences of Echo7 strains shared 80.1 %-80.4 % nucleotide sequence identity. Distance analysis showed that the nucleotide identities of 5′-UTR, P1, P2, P3, 3′-UTR among these strains were 81.4%-85.2%, 77.9 %-81.6 %, 77.6 %-81.3 %, 77.5 %-81.8 %, and 79.0 %-82.3 %, respectively. The three Wallace strains shared more than 99.8 % nucleotide sequence identity in the complete genome.

Thirty-seven reference strains, including prototype strains and modern strains, of enterovirus from the HEV-B group (CAV-9, CVB1 to CVB6, E1 to E7, E9, E11 to E21, E24 to E27, E29 to E33, and EV69 and EV73) were selected from GenBank. CVA16, Polio 1 and EV70 represented HEV-A, HEV-C, HEV-D, respectively. Phylogenetic analysis was carried out separately for the P1, P2, and P3 regions. In the P1 region, all the Echo7 isolates were clustered with Wallace with a high bootstrap value (Fig. 2a). In the P2 region, DH22G/JS/2012 clustered with the CB4 strain (JX308222), which prevailed in China in 2010, with a bootstrap value of 100 %. The Echo7 strain, LR11F7 and UMMC clustered with CB1, Eco9, and Eco19 with a low bootstrap value (Fig. 2b). In the P3 region, DH22G/JS/2012 exhibited a close relationship to the CB4 strain (JX308222) with a bootstrap value 100 %. The Echo7 strain LR11F7 clustered with Eco13, Eco30, and CB4 with a high bootstrap value. The UMMC strain exhibited a close relationship to the CB2 strain that prevailed in Korea in 2006 (Fig. 2c).

**Fig. 2:**
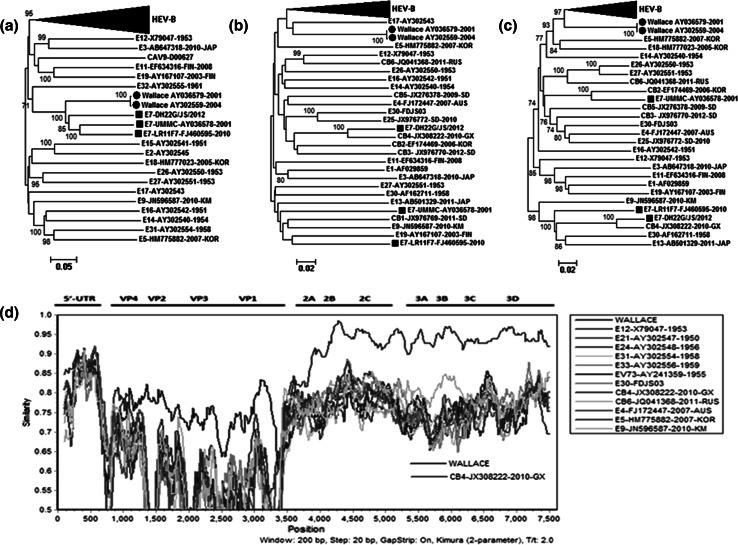
Phylogenetic trees based on nucleotide sequences of Echo7 strains and HEV-B virus. Each of the major functional regions of the genome was analyzed independently. The neighbor-joining method was used to construct the tree. The phylogenetic tree was determined for 1,000 replicates with random seeds. Only strong bootstrap values (>70 %) are shown. A circle indicates Echo7 strains used in the study. a, complete P1 region; b, complete P2 region; c, complete P3 region. d. Simplot analysis based on full-length genomes of the Echo7 and HEV-B strains using a sliding window of 200 nt moving in 20-nt steps. DH22G/JS/2012 showed possible recombination with CVB4 in the P2 and P3 region

The incongruent tree topologies between the structural and nonstructural regions suggested that recombination might have occurred. To investigate this, the complete genome sequences of HEV-B were analyzed with a sliding window of 500 nt, overlapping by 20 nt, using the Simplot program (version 3.5.1) [10]. As shown in Fig. 2d, although the DH22G/JS/2012 strain shared the highest degree of similarity to Wallace in the P1 region (more than 70 %), it had the highest degree of similarity to CB4 in P2 and P3 regions. The similarity between DH22G/JS/2012 and the CB4 strain (JX308222) was greater than 90 % in the P2 and P3 regions.

Although Echo7 infection in association with acute respiratory infection has been reported previously [11], we show here the first case of Echo7 isolated from a HFMD patient in mainland China for which a complete genome sequence has been determined. In addition to this one, only five complete genome sequences of Echo7 are available in the GenBank database. We have not only elucidated the etiology of HFMD but also determined the complete genome sequence of Echo7.

Seventy-three VP1 sequences obtained from global Echo7 strains with a size of more than 600 bp were used to investigate the molecular epidemiology of Echo7 in mainland China. The results showed that subgroup B1 and group C prevailed in mainland China from 1998 to 2012 and that subgroup B2, which circulated in India during 2007-2009 [12], began to circulate in mainland China after 2009. The results indicated that at least three subgroups of Echo7 circulated in mainland China after 2009.

Our results suggest that Echo7 strain DH22G/JS/2012, isolated from an HFMD patient, is a recombinant type B EV with its genome derived from strains CVB4 and Wallace. Currently, besides those for the prototype strains, only two complete genome sequences of Echo7 are available in GenBank. The UMMC strain, reported in Malaysia in 2001, was the first Echo7 isolated from an HFMD patient. Bootscanning and phylogenetic analysis revealed that the UMMC strain was not a recombinant with another enterovirus serotype [5]. Although LR11F7 was a recombinant between EV79 and EV86, it was isolated from sewage of unknown origin [13].

Since the complete sequence of an Echo7 strain from mainland China has never been reported, the recombination of Echo7, including the origins and timelines, is not clear. Most common clinical syndromes associated with coxsackievirus B4 include aseptic meningitis, encephalitis, myopericarditis, neonatal infections, febrile rash, and respiratory manifestations. Coxsackievirus B4 had one of the highest rates of fatal outcome (9.8 %) [2, 14, 15]. Therefore, more studies are needed to understand the pathogenic potential and pathogenic roles of emerging Echo7 strains such as recombinant CVB4.
